# Resveratrol and *N-*acetylcysteine influence redox balance in equine articular chondrocytes under acidic and very low oxygen conditions

**DOI:** 10.1016/j.freeradbiomed.2015.05.008

**Published:** 2015-09

**Authors:** John A. Collins, Robert J. Moots, Peter D. Clegg, Peter I. Milner

**Affiliations:** a Institute of Ageing and Chronic Disease, University of Liverpool, Leahurst Campus, Cheshire, UK, CH64 7TE; b Institute of Ageing and Chronic Disease, University of Liverpool, University Hospital Aintree, Liverpool, UK, L9 7AL

**Keywords:** Cartilage, Acidosis, Oxygen, Redox balance, Antioxidants

## Abstract

Mature articular cartilage is an avascular tissue characterized by a low oxygen environment. In joint disease, acidosis and further reductions in oxygen levels occur, compromising cartilage integrity.This study investigated how acidosis and very low oxygen levels affect components of the cellular redox system in equine articular chondrocytesand whether the antioxidants resveratrol and *N-*acetylcysteine could modulate this system. We used articular chondrocytes isolated from nondiseased equine joints and cultured them in a 3-D alginate bead system for 48 h in <1, 2, 5, and 21% O_2_ at pH 7._2_ or 6.2 in the absence or presence of the proinflammatory cytokine, interleukin-1β (10 ng/ml).In addition, chondrocytes were cultured with resveratrol (10 µM) or *N-*acetylcysteine (NAC) (2 mM).Cell viability, glycosaminoglycan (GAG) release, mitochondrial membrane potential (ΔΨ_m_), reactive oxygen species (ROS), GSH:GSSG ratio, and SOD1 and SOD2 protein expression were measured. Very low levels of oxygen (<1%), acidosis (pH 6.2), and exposure to IL-1β led to reductions in cell viability, increased GAG release, alterations in ΔΨ_m_ and ROS levels, and reduced GSH:GSSG ratio. In addition, SOD1 and SOD2 protein expressions were reduced. Both resveratrol and NAC partially restored ΔΨ_m_ and ROS levels and prevented GAG release and cell loss and normalized SOD1 and SOD2 protein expression. In particular NAC was highly effective at restoring the GSH:GSSG ratio.These results show that the antioxidants resveratrol and *N-*acetylcysteine can counteract the redox imbalance in articular chondrocytes induced by low oxygen and acidic conditions.

## Highlights

• Acidity and low oxygen occur in joint disease.
• We investigated how acidosis and low oxygen affected the redox system in chondrocytes.
• We looked at whether resveratrol and *N*-acetylcysteine could modulate these changes.
• Acidosis and low oxygen significantly altered components of the redox system.
• These changes were defended in the presence of antioxidants.

## Introduction

Articular cartilage allows frictionless and pain-free movement in joints [1].The avascular and relatively hypoxic nature of mature articular cartilage, however, provides a challenging environment for the resident cell, the chondrocyte, to function [2]. Chondrocytes are responsible for the production and maintenance of cartilage extracellular matrix consisting of collagens, proteoglycans, glycosaminoglycans, noncollagenous proteins, and water [3].Although being considered relatively metabolically inactive (due to the absence of a vascular supply) chondrocytes respond to mechanical stimuli, growth factors, and cytokines by altering matrix production and their survival is key to cartilage integrity since chondrocytes are not replaced if damaged [4].

Oxygen levels in cartilage are estimated to be 10% at the surface, decreasing down a diffusion gradient to around 2% in deeper levels [5]. Consequently, energy production in chondrocytes is predominately through substrate-level phosphorylation with the production of lactic acid, although some oxidative phosphorylation is thought to occur in superficial levels of cartilage [6].In addition, the fixed negative charge from the proteoglycan network leads to increased matrix cation concentrations, including Na^+^ and H^+^. Chondrocytes therefore experience both inward H^+^ leak and intracellular acid loading [7]. Despite this constant acid challenge, articular chondrocytes are able to maintain their resting intracellular pH at approximately 7.1 through the activity of Na^+^/H^+^ exchanger (NHE), H^+^-ATPase, and relatively high intracellular buffering capacity (around 30 mmol L^–1^(pH^–1^) [8–12].

The importance of oxygen and pH in articular cartilage as modulators of chondrocyte activity is being increasingly recognized [13]. Optimal matrix production occurs around 5% O_2_ with very low levels affecting cell viability and matrix synthesis [14].Chondrocyte gene expression shows pH-sensitivity and changes in extracellular and intracellular pH result in a bimodal response of matrix production [15,16].In diseases such as osteoarthritis and rheumatoid arthritis, further reductions in oxygen and pH occur [17–19].In this environment, release of inflammatory mediators, such as interleukin-1 (IL-1), also results in altered chondrocyte gene expression and release of degradative enzymes (matrix metalloproteinases), leading to cartilage breakdown and hence loss of joint function [20].Chondrocytes also produce reactive oxygen and nitrogen species (RONS) in response to changes in oxygen, mechanical stress, and inflammatory mediators and possess enzymatic and nonenzymatic systems to regulate RONS levels and alterations in RONS levels have been linked to matrix production and cell survival in chondrocytes [21]. In addition, reductions in antioxidant enzymes, such as superoxide dismutases, are linked to naturally occurring and animal models of osteoarthritis [22,23].

Despite the fundamental relationship of oxygen and pH with redox reactions little is known about how changes in oxygen and pH levels may affect redox balance in normal articular chondrocytes, although work studying chondrocytes from osteoarthritic cartilage has been recently published [24].It is likely that factors regulating redox balance will impact on chondrocyte function and survival and therefore be important to cartilage integrity *in situ* and tissue bioengineering [15,25].Furthermore, the effect of exogenous antioxidants on these redox processes under low oxygen and pH conditions is not well documented in cartilage.Resveratrol and *N-*acetylcysteine (NAC) have been shown to possess chondroprotective properties that may be linked with redox balance in experimental models of osteoarthrtitis [26–28].The aims of this study were to characterize the redox changes of equine articular chondrocytes when cultured in very low oxygen and low pH conditions and in the presence of the proinflammatory cytokine, IL-1, and to assess their response to the antioxidants resveratrol and NAC.

## Materials and methods

### Chondrocytes isolation and culture conditions

All chemicals were from Sigma Aldrich (UK) unless specified. Full thickness cartilage slices were dissected from the metacarpophalangeal joint of skeletally mature horses (*n*=18) euthanized at a commercial abattoir. Tissue samples for this study were collected as a by-product of the agricultural industry. In this respect, the Animal (Scientific Procedures) Act 1986, Schedule 2, does not define collection from these sources as scientific procedures and so ethical approval was not required for this study.Cartilage was only used from joints with macroscopically normal cartilagescore of zero (no signs of surface roughening, fibrillations, or erosions) based on a modified scoring system [29]. Slices were mixed randomly for each individual horse and suspended in Dulbecco׳s modified Eagles medium (DMEM) minus Phenol Red and sodium bicarbonate supplemented with 10% fetal calf serum (FCS), 100 units/ml penicillin, 100 µg/ml streptomycin, and 500 ng/ml amphotericin B and buffered with 25 mM 4-(2-hydroxyethyl)-1-piperazineethanesulfonic acid (Hepes). Chondrocytes were released from cartilage matrix by standard enzymatic digestion (collagenase type II 0.8 mg/ml, 37 °C, 15 h). Cell viability was determined by trypan blue exclusion and was >95% for all samples [30].Primary chondrocytes were then cultured directly into three-dimensional alginate beads. Cells were suspended in low viscosity (1.2%) alginate solution at a cell density of 4×10^6^/ml. The alginate/cell suspension was released dropwise into petri dishes containing CaCl_2_ (102 mM). Following polymerization, beads were washed twice in NaCl (150 mM) and incubated in DMEM (minus Phenol Red and sodium bicarbonate plus 10% FCS) with 25 mM Hepes (pH 7.2) for 14 days at 5% O_2_. Aliquots of cells were taken after the 14 day stabilization period to assess articular chondrocyte phenotype [31,32].

The remaining alginate beads were incubated in DMEM (minus Phenol Red and sodium bicarbonate plus 10% FCS) with 25 mMHepes (pH 7.2) or 10 mM piperazine-*N-N*′-bis(2-ethanesulfonic acid) (Pipes) (pH 6.2) in the presence or absence of IL-1β (10 ng/ml) and in the presence or absence of resveratrol (10 µM) or *N-*acetylcysteine (2 mM) for 48 h in <1, 2, 5, or 21% O_2_ (all 5% CO_2_, 37 °C). The concentrations chosen for *N-*acetylcysteine and resveratrol were based on previous studies in chondrocytes [27,33].Media were equilibrated prior to incubation to the appropriate oxygen tension, confirmed by an oxygen electrode (Oxyview,-1, Hanstech, UK). For <1% O_2_ conditions, the oxygen scavenger sodium dithionite (1.5 mM) was added to the media. Following 48 h incubation, chondrocytes were released from their alginate matrix by depolymerization (sodium citrate, 55 mM),centrifuged, (1400 rpm) andthenwashed in sterile phosphate buffered saline (PBS) (equilibrated to required oxygen tension) prior to analysis. Media from each sample were frozen (−20 °C) for later analysis.

### Live/dead cell assay

Cell death was assessed through release of lactate dehydrogenase (LDH) into media and quantified using the Cytotoxicity Detection Kit^Plus^ as per the manufacturer׳s instructions (Roche Diagnostics Ltd, UK). Briefly, 100 µl of media from each sample was added (in triplicate) to a 96-well plate before adding 100 µl of reaction mixture (30 min, 20 °C) before dual absorbance measurements (490 and 600 nm).

### Glycosaminoglycan release

Glycosaminoglycan (GAG) concentrations in media were measured using the dimethylmethylene blue (DMMB) assay [34].A standard curve was generated using chondroitin sulphate C as the standard. In triplicate, 40 µl of each standard and 40 µl from each media sample were added to a 96-well plate. From a stock solution of DMMB dye, 250 µl was added to each unknown sample and standard. Absorbance was immediately read at 570 nm.GAG concentrations from media samples were determined using the standard curve.

### Mitochondrial membrane potential

Mitochondrial membrane potential (ΔΨ_m_) was measured using 5,5′,6,6′-tetrachloro-1,1′,3,3′ tetraethylbenzimidazolylcarbocyanine iodide (JC-1) [35]. Following incubation, chondrocytes were loaded with JC-1 (50 µg/ml) in <1, 2, 5, or 21% O_2_ (37 °C, 40 min) and then washed twice in PBS (preequilibrated at relevant oxygen level) before aliquots of cells were fixed on cover slides (Vector Laboratories, Peterborough, UK). Red and green fluorescence intensity (Ex=490 nm; Em=585 nm for red and Em=514 nm for green) was measured using a Nikon Eclipse TS-100 inverted microscope with Eclipse TS-100/TS-100-F fluorescence attachment (Nikon, Surrey, UK) and intensity ratio calculated using Image J 1.42 software. A reduction in the ratio indicates a mitochondrial membrane depolarization.

### Intracellular reactive oxygen species (ROS) levels

Intracellular ROS levels were measured using the dichlorofluorescin diacetate (DCF-DA) method [36].After incubation chondrocytes were loaded with 10 µM DCF-DA in <1, 2, 5, or 21% O_2_ (37 °C, 40 min). Cells were then washed twice in PBS (preequilibrated at relevant oxygen level). DCF oxidation was assessed spectrophotometrically by DCF emission (Ex=490 nm; Em=535 nm).

### Intracellular GSH:GSSG ratio

Total glutathione and oxidized glutathione (GSSG) was measured using GSH:GSSG-Glo assay (Promega, Hants, UK) as per manufacturer׳s instructions. From total glutathione and oxidized gluthathione, reduced glutathione (GSH) was determined and hence GSH:GSSG ratio calculated.

### Protein expression analysis by Western blotting

Cell lysates (10 µg protein) were separated on Nu-Page 4–12% SDS-PAGE gels (Life Technologies, Paisley, UK), transferred to a nitrocellulose membrane, andthen blocked using PBS/Tween bufferand nonfat dried milk (w/v 1%) solution for 1 h. Following this membranes were incubated overnight (15 h, 4 °C) with primary antibodies versus superoxide dismutase 1 (SOD1, 1:1000) (Cat. No.ADI-SOD-100, Enzo Life Sciences, Devon, UK), superoxide dismutase 2 (SOD2, 1:1000) (Cat. No. ADI-SOD-111, Enzo Life Sciences, Devon, UK), COL2A1 (1:1000) (Cat. No. sc-7764, Santa Cruz Biotechnology, Hiedelberg, Germany) or SOX9 (1:1000) (Cat. No.AB5535 Millipore, Herts, UK) with α-tubulin (1:1000) (Cat. No.ab4074, Abcam, Cambs, UK) used as loading control. Membranes were washed thrice using PBS/Tween and incubated with horseradish peroxidise (HRP)-conjugated secondary antibody (1:2000, SourceBioscience, Notts, UK) (1 h, 20 °C) with bands detected using a chemiluminescence detection kit (Western Lightning Plus, Bucks, UK). Bands were imaged using the VisionWorksLS image acquisition software package and band densities were analyzed using ImageJ 1.42 software and normalized to loading control.

### Statistical analysis

Data were analyzed using IBM SPSS Statistics 20. Normal distribution for each data set was assessed using the Kolmogorov-Smirnov test for normality. All data sets were normally distributed. Statistical analysis was performed on all data sets and significant differences were determined by one-way ANOVA with a Dunnett׳s and Tukey HSD post hoccorrection. Results are presented as mean values±SEM with each *n* value being performed in triplicate. Exact *P*values are presented for all data sets as appropriate.A minimal significance level of *P*<0.05 was used. Unless otherwise stated, results are presented as percentage compared to control (5% O_2_, pH7.2, time=0).

## Results

### Cell viability, articular chondrocyte phenotype, and glycosaminoglycan release

**Fig. 1:**
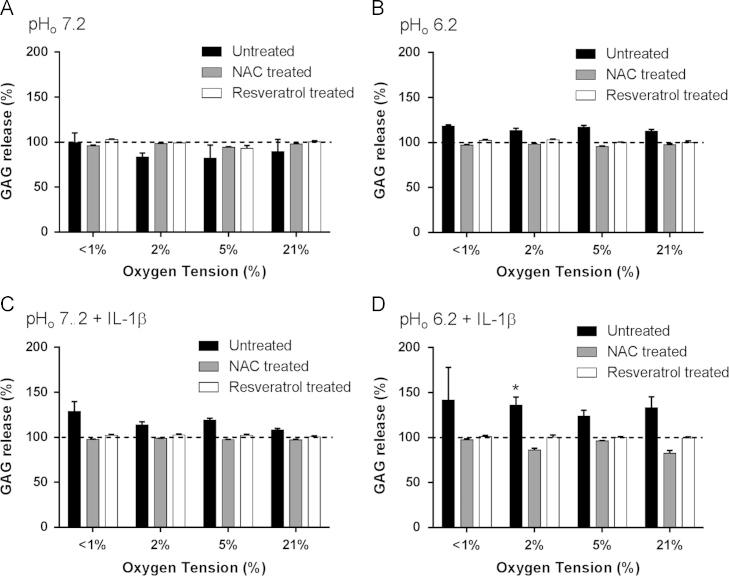
Effect of oxygen tension, pH, and IL-1β on glyosaminoglycan (GAG) release from equine articular chondrocytes in the absence or presence of resveratrol or *N-*acetylcysteine. Equine articular chondrocytes were cultured in 3D alginate beads for 48 h in <1, 2, 5, or 21% O_2_ at pH 7.2 (A), pH 6.2 (B), pH 7.2 plus 10 ng/ml IL-1β (C) or pH 6.2 plus 10 ng/ml IL-1β (D) in the absence or presence of *N-*acetylcysteine (2 mM) or resveratrol (10 µM). GAG release was measured in media using the dimethylmethylene blue (DMMB) assay. Bar charts represent mean±SEM, *n*=3. *P<0.05 versus control (time=0, 5%O_2_, pH 7.2).

Western blot analysis showed no change in expression of COL2A1 and SOX9 protein after the culture period, indicating maintenance of chondrocyte phenotype during this study (Fig. S1). Incubation in <1% O_2_ showed significant increase in LDH release, indicating reduced cell viability (Fig S2). In the presence of resveratrol (10 µM) or NAC (2 mM) this was reduced to control levels. Glycosaminoglycan release was significantly increased under acidic (pH 6.2) conditions in low oxygen (2% O_2_) in the presence of IL-1β(10 ng/ml) (136.2±4.8%, *P*= 0.001) (Fig. 1).Similarly, this increase was abatedwhen chondrocytes were incubated with resveratrol or NAC.

### Effect of pH, oxygen,and interleukin-1 on mitochondrial membrane potential and ROS levels in equine articular chondrocytes

**Fig. 2:**
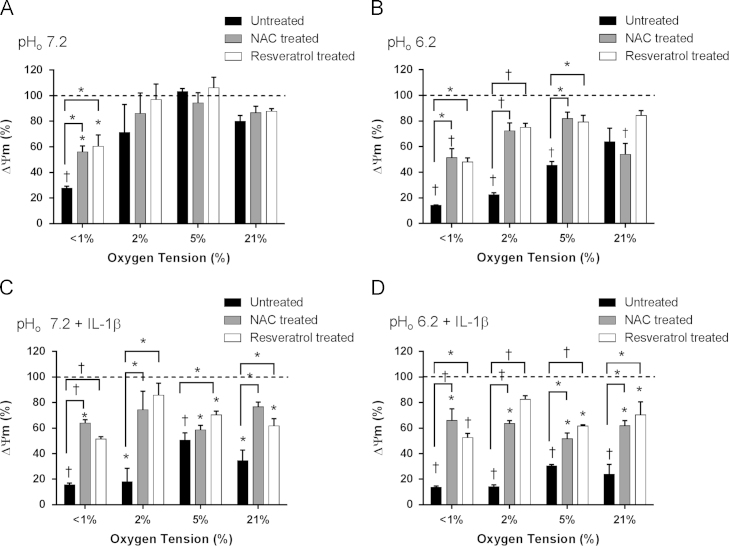
Effect of oxygen tension, pH, and IL-1β on mitochondrial membrane potential (ΔΨ_m_) in equine articular chondrocytes in the absence or presence of resveratrol or *N-*acetylcysteine. Equine articular chondrocytes were cultured in 3D alginate beads for 48 h in <1, 2, 5, or 21% O_2_ at pH 7.2 (A), pH 6.2 (B), pH 7.2 plus 10 ng/ml IL-1β (C) or pH 6.2 plus 10 ng/ml IL-1β (D) in the absence or presence of *N-*acetylcysteine (2 mM) or resveratrol (10 µM). ΔΨ_m_ was measured using the fluorescent probe JC-1. Bar charts represent mean±SEM, *n*=3. *P<0.05;^†^*P*<0.01 versus control (time=0, 5%O_2_, pH 7.2) or between groups where shown.

Acidosis (pH 6.2) and reduced oxygen levels (<1% O_2_) led to significant mitochondrial membrane depolarization(Fig. 2A and B). In the presence of resveratrol in acidosis (pH 6.2) there was less depolarization,although levels were still below control conditions.Additionally, under very low oxygen conditions (<1% O_2_) ΔΨ_m_ increased from 27±2 to 65±9% in the presence of resveratrol (*P* =0.004). NAC similarly reduced hypoxic-dependent depolarization by significantly increasing ΔΨ_m_ under acidic conditions (at <1, 2, and 5% O_2_, pH 7.2) but had no effect at 21% O_2_. At very low oxygen levels (<1% O_2_, pH 7.2),*N-*acetylcysteine had an effect similarto that of resveratrol with ΔΨ_m_ levels increased to 56±5% of control values (*P*= 0.002).IL-1β had a pronounced effect on mitochondrial membrane potential, irrespective of oxygen level or pH (Fig. 2C and D). Both resveratrol and NAC reduced the level of mitochondrial membrane depolarization caused by IL-1β but levels still remained below control values.

**Fig. 3:**
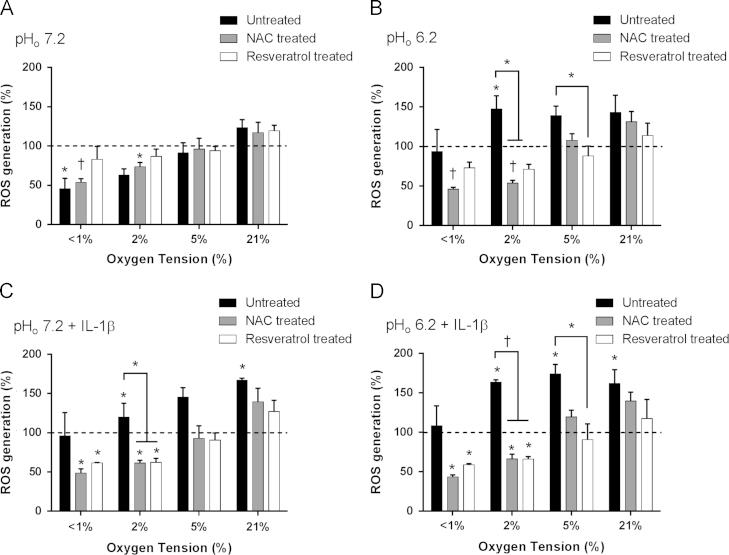
Effect of oxygen tension, pH, and IL-1β on reactive oxygen species levels in equine articular chondrocytes in the absence or presence of resveratrol or *N-*acetylcysteine. Equine articular chondrocytes were cultured in 3D alginate beads for 48 h in <1, 2, 5, or 21% O_2_ at pH 7.2 (A), pH 6.2 (B), pH 7.2 plus 10 ng/ml IL-1β (C) or pH 6.2 plus 10 ng/ml IL-1β (D) in the absence or presence of *N-*acetylcysteine (2 mM) or resveratrol (10 µM). ROS levels were measured using the fluorescent probe DCF-DA. Bar charts represent mean±SEM, *n*=3. *P<0.05; ^†^*P*<0.01 versus control (time=0, 5%O_2_, pH 7.2) or between groups where shown.

ROS levels were reduced in very low oxygen levels (<1% O_2_, pH 7.2) (*P*=0.02) but increased under acidic conditions (pH 6.2) (*P*=0.04) (Fig. 3A and B). ROS levels were significantly increased in the presence of IL-1β under all oxygen conditions (Fig. 3C and D). The reduction in ROS levels present under very low oxygen conditions was prevented by the presence of resveratrol and similarly resveratrol prevented the acid-induced increase in ROS. NAC did not appear to affect ROS levels under low oxygen conditions but muted the acid-induced rise when incubated in 2%O_2_.Both resveratrol and NAC reduced the IL-1β induced increase in ROS levels.

### Effect of pH, oxygen, and interleukin-1 on reduced/oxidized glutathione (GSH:GSSG) ratio in equine articular chondrocytes

**Fig. 4:**
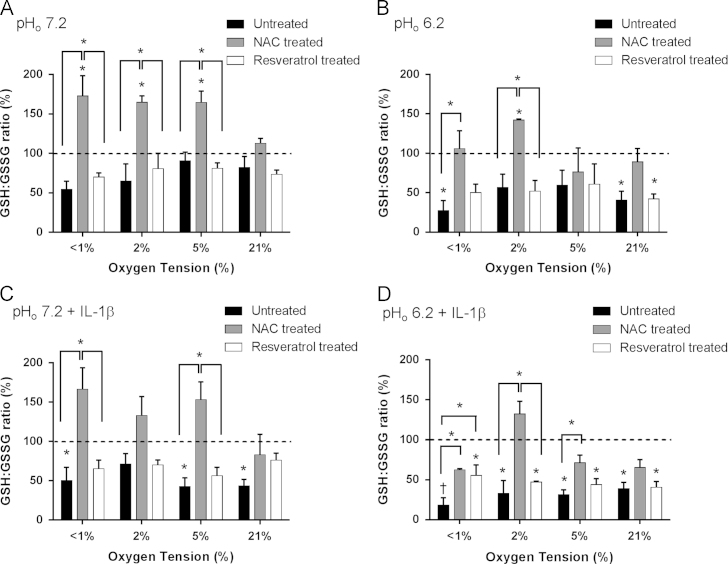
Effect of oxygen tension, pH, and IL-1β on GSH:GSSG ratio in equine articular chondrocytes in the absence or presence of resveratrol or *N-*acetylcysteine. Equine articular chondrocytes were cultured in 3Dalginate beads for 48 h in <1, 2, 5, or 21% O_2_ at pH 7.2 (A), pH 6.2 (B), pH 7.2 plus 10 ng/ml IL-1β (C) or pH 6.2 plus 10 ng/ml IL-1β (D) in the absence or presence of *N-*acetylcysteine (2 mM) or resveratrol (10 µM). GSH:GSSG ratio was calculated using the GSH/GSSG-Glo assay. Bar charts represent mean±SEM, *n*=3. *P<0.05; ^†^*P*<0.01 versus control (time=0, 5%O_2_, pH 7.2) or between groups where shown.

The ratio of reduced (GSH) to oxidized glutathione (GSSG) is an indicator of cellular redox status. GSH:GSSG ratio was significantly reduced under acidic conditions under very low oxygen conditions (<1% O_2_, pH 6.2) (*P*=0.004) as well as under acidic conditions at 21% O_2_(*P*=0.004) (Fig. 4A and B). At 2 and 5% O_2_ (pH 6.2), GSH:GSSG ratio was reduced but this was not significant. IL-1β markedly reduced GSH:GSSG ratio under all conditions (Fig. 4C and D). Interestingly NACsignificantly increased GSH:GSSG ratio above control levels, particularly in pH 7.2 and negated the effects of acidosis and low oxygen particularly in the presence of IL-1β. Resveratrol had little effect on GSH:GSSG ratio under acidic or low oxygen conditions in the presence or absence of IL-1β. Changes in GSH:GSSG ratio were primarily due to alterations in cellular GSH levels (Fig. S3).

### SOD1 and 2 protein expression in equine articular chondrocytes

**Fig. 5:**
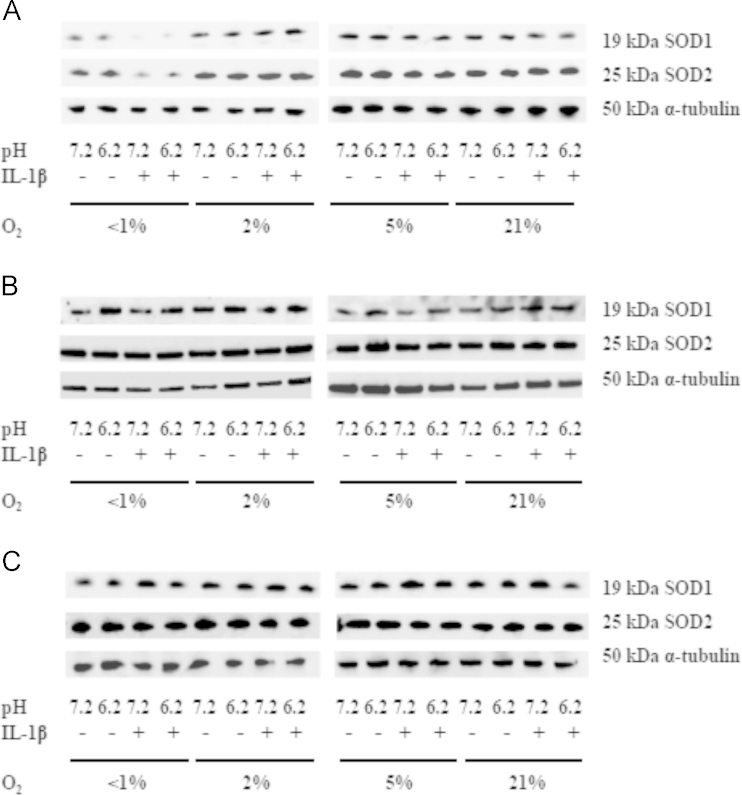
Representative Western blots showing the effect of oxygen tension, pH, and IL-1β on protein expression of superoxide dismutase 1 (Cu/ZnSOD, SOD1), superoxide dismutase 2 (MnSOD, SOD2), and α-tubulin from equine articular chondrocytes. Equine articular chondrocytes were cultured in 3D alginate beads for 48 h in <1, 2, 5, or 21% O_2_ at pH 7.2 or pH6.2 in the presence or absence of 10 ng/ml IL-1β (A), in the presence of resveratrol (10 µM) (B) or *N-*acetylcysteine (2 mM) (C).

Expression of SOD1 was significantly decreased under very low oxygen conditions (<1% O_2_) at both pH 7.2 and 6.2 (*P*=0.03 and 0.01, respectively) and in the presence of IL-1β (*P*=0.002 and 0.002, respectively) (Fig. 5A and Fig. S4A–D).IL-1β also significantly reduced SOD2 protein expression under very low oxygen conditions at pH 7.2 and 6.2 (*P*=0.036 and 0.024, respectively) (Fig. 5A and Fig. S5C and D).Both resveratrol and NAC restored SOD1 and 2 protein expression under very low oxygen conditions (<1% O_2_) and in the presence of IL-1β (Fig. 5B and C).

## Discussion

Redox imbalance is an important component in the pathophysiology of a number of conditions such as diabetes, neurodegeneration, cardiac disease, and cancer.There is increasing evidence that redox imbalance features in joint diseases such asrheumatoid arthritis (RA) and osteoarthritis (OA) [22,23,37]as well as being involved in age-related degeneration of articular cartilage [21].Hypoxia, acidosis,and the release of proinflammatory cytokines are also recognized features of RA and OA [17–19]. Since oxygen and pH are fundamental components of chemical redox reactions [38] and redox imbalance is reported in joint disease the primary focus of this study was to determine how these external parameters affect the processes involved in cellular redox balance and whether these can be manipulated with exogenous compounds. This study used articular chondrocytes in a 3-D alginate model under conditions of low oxygen and acidosis and in the presence of the proinflammatory cytokine, IL-1β, to investigate the effects of two antioxidants, resveratrol and NAC, on components of the cellular redox system. We show that low oxygen, acidosis, and IL-1β lead to cell death, glycosaminoglycan loss, mitochondrial membrane depolarization, alterations in reactive oxygen species (ROS) levels, reductions in reduced/oxidized glutathione (GSH:GSSG) ratio, and reduced expression of superoxide dismutase (SOD) 1 and 2 in articular chondrocytes. Further we show how these parameters are modulated by the antioxidants resveratrol and NAC.This study provides support for the potential use of these compounds as disease-modifying drugs in the progressive stages of joint disease [26,28].

Weused articular chondrocytes isolated from grossly normal equine joints; this source provides a suitable model for studying cartilage biology in health and disease [39] and since primary cells are available in large numbers, the potential dedifferentiation step of monolayer expansion is removed [40]. Adopting a 3-D alginate culture system allows for a maintenance of a chondrocytic phenotype and this was supported in this study by the unaltered expression of COL2A1 and SOX9 [29].We chose tissue samples from nondiseased joints to avoid the difficulties of heterogeneity encountered in joint disease and to detect early responses in chondrocytes which may be lost in chronic, end-stage diseased cartilage.

We found that low oxygen levels, acidosis, and the presence of the proinflammatory cytokine IL-1β resulted in a significant increase in cell death and glycosaminoglycan loss from chondrocytes. The harsh environment of very low oxygen and pH has been recorded in diarthrodial joints *in situ* [41,42] and IL-1 is widely recognized as a pivotal cytokine in joint disease [20]. Studying the effects of these parameters is often performed in isolation but the interactions of these components, albeit difficult to perform, are important to consider when translating *in vitro* findings to *in vivo* observations.In this study we show that acidosis (pH 6.2) in particular led to significant mitochondrial membrane depolarization andincrease in ROS generation under hypoxic conditions, compared to pH 7.2.We have previously shown that complex III in the electron transport chain is the main ROS in articular chondrocytes [43] and that the inhibition of the Q cycle at different levels under hypoxic conditions can result in either increased or decreased ROS generation [44]. Reduced formation of semiquinone at the Q_o_ site (*as per*the mechanism of action of myxothiazol) will reduce formation of ROS whereas reduction in the transfer of electrons from the Q_o_ to Q_i_site (*as per*the mechanism of action of antimycin A) results in an accumulation of unstable semiquinone and hence increased ROS levels. We hypothesize that acidosis could act through the latter process, resulting ina reduction in mitochondrial membrane potential and an increase in ROS levels,although we recognize that other, nonmitochondrial sources of ROS, such as membrane-bound NAPDH oxidase, exist within the cell and may be influenced by acidosis.In addition, the oxygen sensitivity of mitochondrial ROS generation is dependent on metabolic conditions [45] and changes in substrate availability to mitochondrial complexes may also be pH sensitive [46]. Since cartilage matrix synthesis is pH dependent [16] and synovial fluid acidosis has been linked to cartilage destruction [47], the importance of extracellular pH on chondrocyte function has become increasingly recognized [15]. The effect of oxygen, particularly hypoxia in cartilage, has received significantly more attention. However, it is important that the levels of oxygen studied are clearly stated, since hypoxia has been used to describe 1, 2, and 5% O_2_.Chondrocytes *in situ* reside in low oxygen in cartilage (around 2–10%) relying on diffusion down a gradient [5] and are adapted to this environment with optimal matrix production occurring between 5 and 10% [25].Although being able to survive in low oxygen, very low oxygen levels can result in chondrocyte apoptosis [32].

As well as the effects on mitochondrial membrane potential, very low levels of oxygen led to decreased protein expression of superoxide dismutase (SOD) 1 and 2. Superoxide dismutases form part of the cellular antioxidant system, along with nonenzymatic components, such as glutathione and thioredoxin, tasked to maintain cellular redox status. SOD1 (Cu/Zn SOD) is predominately located in the cytoplasm, SOD2 (MnSOD) is located in mitochondria, and an extracellular isoform (SOD3) also exists. Downregulation of antioxidant enzymes, such as SOD2, has been reported in osteoarthritic cartilage [23] and linked to mitochondrial dysfunction [48]. Reduced SOD activity under low oxygen conditions may increase susceptibility to cytokine-driven cartilage damage.

Acidosis and exposure to IL-1β resulted in significant reduction in GSH:GSSG ratio. Although not the only nonenzymatic cellular antioxidant, GSH:GSSG can give an indication of the redox status of the cell. Changes in the GSH:GSSG ratio were primarily due to altered GSH levels with GSSG levels unaffected (data not shown). It was not surprising that GSH levels were significantly increased in the presence of NAC but not resveratrol since the primary role of the former is to replenish the cysteine pool and enhance GSH synthesis. This replenishment may explain the effect of NAC on reducing ROS levels, although direct ROS scavenging has also been described [49] and NAC has also been shown to have antiapoptotic properties [27].GSH:GSSG levels have also been linked to mitochondrial dysfunction [50]. Interestingly resveratrol had no effect on GSH:GSSG ratio, although it did reduce ROS levels and partially restore mitochondrial membrane potential. Resveratrol is a naturally occurring polyphenol present in grape skin, red wine, berries, and peanuts and has antiapoptotic properties as well as inhibiting matrix metalloproteinase release [51] and maintaining extracellular matrix integrity [26]. One of its main roles is to maintain mitochondrial membrane potential and scavenge ROS [33].In this study, partial restoration of the mitochondrial membrane potential may be sufficient to allow electron flow through the electron transport chain and hence reduce mitochondrially derived ROS generation,explaining its antioxidant action,although direct ROS scavenging may also be responsible.Resveratrol also acts through sirtuins in regulating apoptosis and cell survival [52].Mice deficient in SIRT-1 show reduced survival and altered chondrocyte phenotype [53] and SIRT1 overexpression can abrogate IL-1β-induced gene expression in articular chondrocytes [54].

## Conclusion

In conclusion the antioxidants resveratrol and *N-*acetylcysteine reduced cell death and glycosaminoglycan loss in chondrocytes cultured under low oxygen and acidic conditions.Both antioxidants worked through restoring mitochondrial membrane potential and normalization of ROS levels and expression of antioxidant enzymes SOD 1 and 2. *N-*Acetylcysteine in particular potently enhanced the cellular GSH:GSSG ratio. This study demonstrated the effectiveness of two antioxidants in counteracting redox imbalance under conditions encountered in joint diseasebymodulating the components involved in cellular antioxidant status.

## Conflict of interest statement

The authors have no conflict of interest to report

## Appendix A Supplementary materials

Supplementary Material: Figure S1. Representative Western blots showing COL2A1, SOX9 and α-tubulin protein expression from equine articular chondrocytes. Equine articular chondrocytes were cultured in 3-D alginate beads for 14 days (5%O_2_, pH 7.2) (**A**) or after further 48 hours at <1%, 2%, 5% or 21% O_2_ (pH 7.2) (**B**). Band densitometry was used to quantify protein expression in chondrocytes cultured in pH 7.2 (**C**) or pH 6.2 (**D**). Data were normalised to α-tubulin and expressed as percentage of control (time = 0, 5% O_2_, pH 7.2). Bar charts represent mean ± SEM, n=3.

Supplementary Material: Figure S2. Effect of oxygen tension, pH and IL-1β on lactate dehydrogenase (LDH) release from equine articular chondrocytes in the absence or presence of resveratrol or N-acetylcysteine. Equine articular chondrocytes were cultured in 3D-alginate beads for 48 hours in <1%, 2%, 5% or 21% O_2_ at pH 7.2 (**A**), pH 6.2 (**B**), pH 7.2 plus 10 ng/ml IL-1β (**C**) or pH 6.2 plus 10 ng/ml IL-1β (**D**) in the absence or presence of N-acetylcysteine (2 mM) or resveratrol (10 µM). LDH release was measured in media using the Cytotoxicity Detection Kit^Plus^. Bar charts represent mean ± SEM, n=3. **P*<0.05 versus control (time = 0, 5%O_2_, pH 7.2).

Supplementary Material: Figure S3. (**A**) Reduced (GSH) and (**B**) oxidised glutathione (GSSG) levels in equine articular chondrocytes. Equine articular chondrocytes were cultured in 3D-alginate beads for 48 hours in <1%, 2%, 5% or 21% O_2_ at pH 7.2 or pH6.2 in the presence or absence of 10 ng/ml IL-1β and in the presence of resveratrol (10 µM) or N-acetylcysteine (2 mM). Data are presented as percentage luminescence of control (time = 0, 5% O_2_, pH 7.2). Bar charts represent mean ± SEM, n=3.**P*<0.05 versus control.

Supplementary Material: Figure S4. Densitometric analysis of SOD1 protein expression from equine articular chondrocytes. Equine articular chondrocytes were cultured in 3D-alginate beads for 48 hours in <1%, 2%, 5% or 21% O_2_ at pH 7.2 (**A**) or pH6.2 (**B**) in the presence or absence of 10 ng/ml IL-1β (**C**, **D**) in the presence of resveratrol (10 µM) or N-acetylcysteine (2 mM). Data are normalised to α-tubulin and expressed as percentage of control (time = 0, 5% O_2_, pH 7.2). Bar charts represent mean ± SEM, n=3.**P*<0.05 versus control.

Supplementary Material: Figure S5. Densitometric analysis of SOD2 protein expression from equine articular chondrocytes. Equine articular chondrocytes were cultured in 3D-alginate beads for 48 hours in <1%, 2%, 5% or 21% O_2_ at pH 7.2 (**A**) or pH6.2 (**B**) in the presence or absence of 10 ng/ml IL-1β (**C**, **D**) in the presence of resveratrol (10 µM) or N-acetylcysteine (2 mM). Data are normalised to α-tubulin and expressed as percentage of control (time = 0, 5% O_2_, pH 7.2). Bar charts represent mean ± SEM, n=3.**P*<0.05 versus control.
