# Supplementary figures and images for: Resveratrol and N-acetylcysteine influence redox balance in equine articular chondrocytes under acidic and very low oxygen conditions
Source: Free Radic Biol Med. 2015 Sep;86:57–64. doi: 10.1016/j.freeradbiomed.2015.05.008 (PMC4562226; doi:10.1016/j.freeradbiomed.2015.05.008)

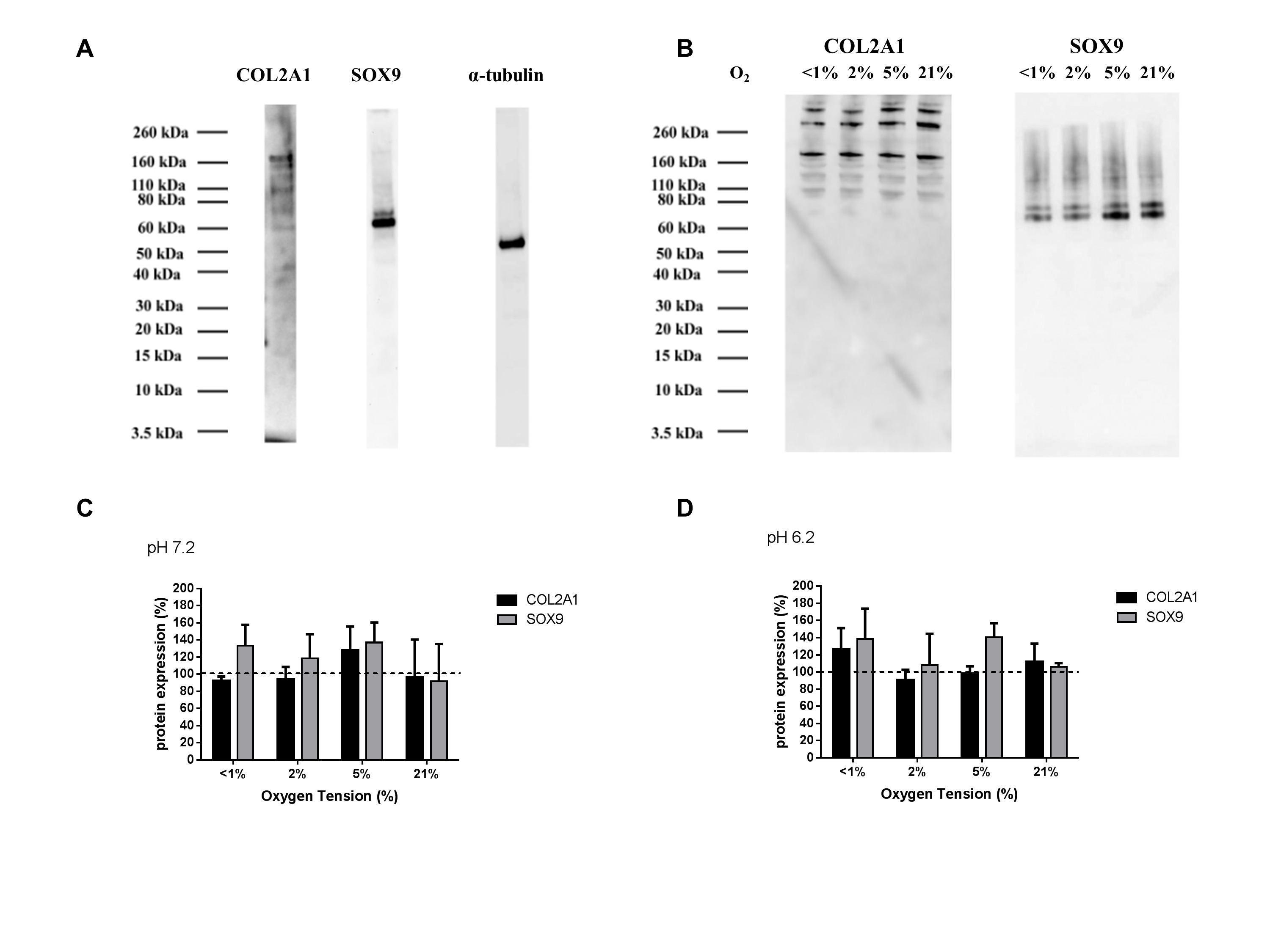

Supplement: Supplementary file 1 — Supplementary Material: Figure S1. Representative Western blots showing COL2A1, SOX9 and α-tubulin protein expression from equine articular chondrocytes. Equine articular chondrocytes were cultured in 3-D alginate beads for 14 days (5%O2, pH 7.2) (A) or after further 48 hours at <1%, 2%, 5% or 21% O2 (pH 7.2) (B). Band densitometry was used to quantify protein expression in chondrocytes cultured in pH 7.2 (C) or pH 6.2 (D). Data were normalised to α-tubulin and expressed as percentage of control (time = 0, 5% O2, pH 7.2). Bar charts represent mean ± SEM, n=3. [file mmc1.zip › Figure S1.tif]

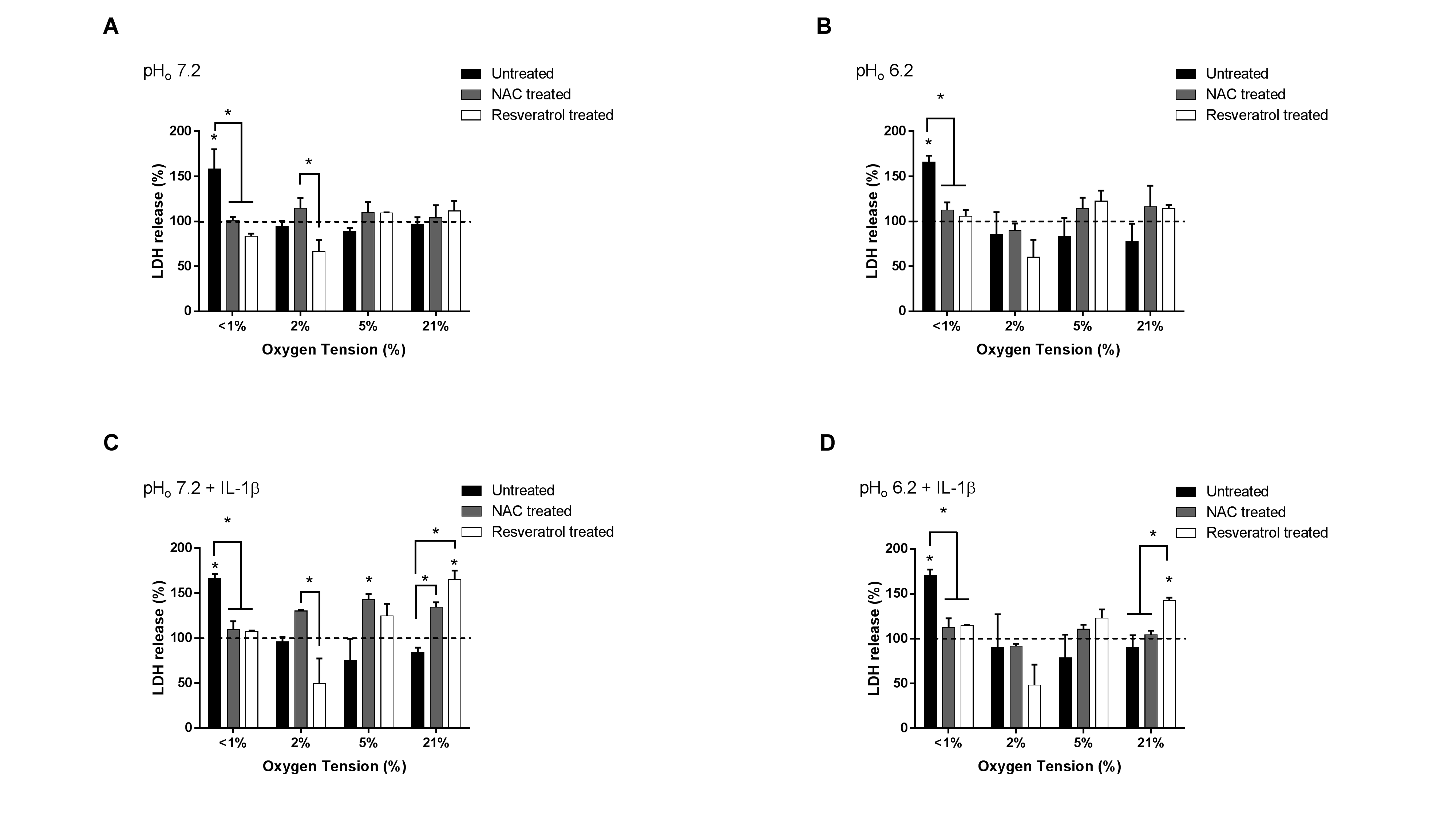

Supplement: Supplementary file 2 — Supplementary Material: Figure S2. Effect of oxygen tension, pH and IL-1β on lactate dehydrogenase (LDH) release from equine articular chondrocytes in the absence or presence of resveratrol or N-acetylcysteine. Equine articular chondrocytes were cultured in 3D-alginate beads for 48 hours in <1%, 2%, 5% or 21% O2 at pH 7.2 (A), pH 6.2 (B), pH 7.2 plus 10 ng/ml IL-1β (C) or pH 6.2 plus 10 ng/ml IL-1β (D) in the absence or presence of N-acetylcysteine (2 mM) or resveratrol (10 µM). LDH release was measured in media using the Cytotoxicity Detection KitPlus. Bar charts represent mean ± SEM, n=3. *P<0.05 versus control (time = 0, 5%O2, pH 7.2). [file mmc2.zip › Figure S2 LDH release.tif]

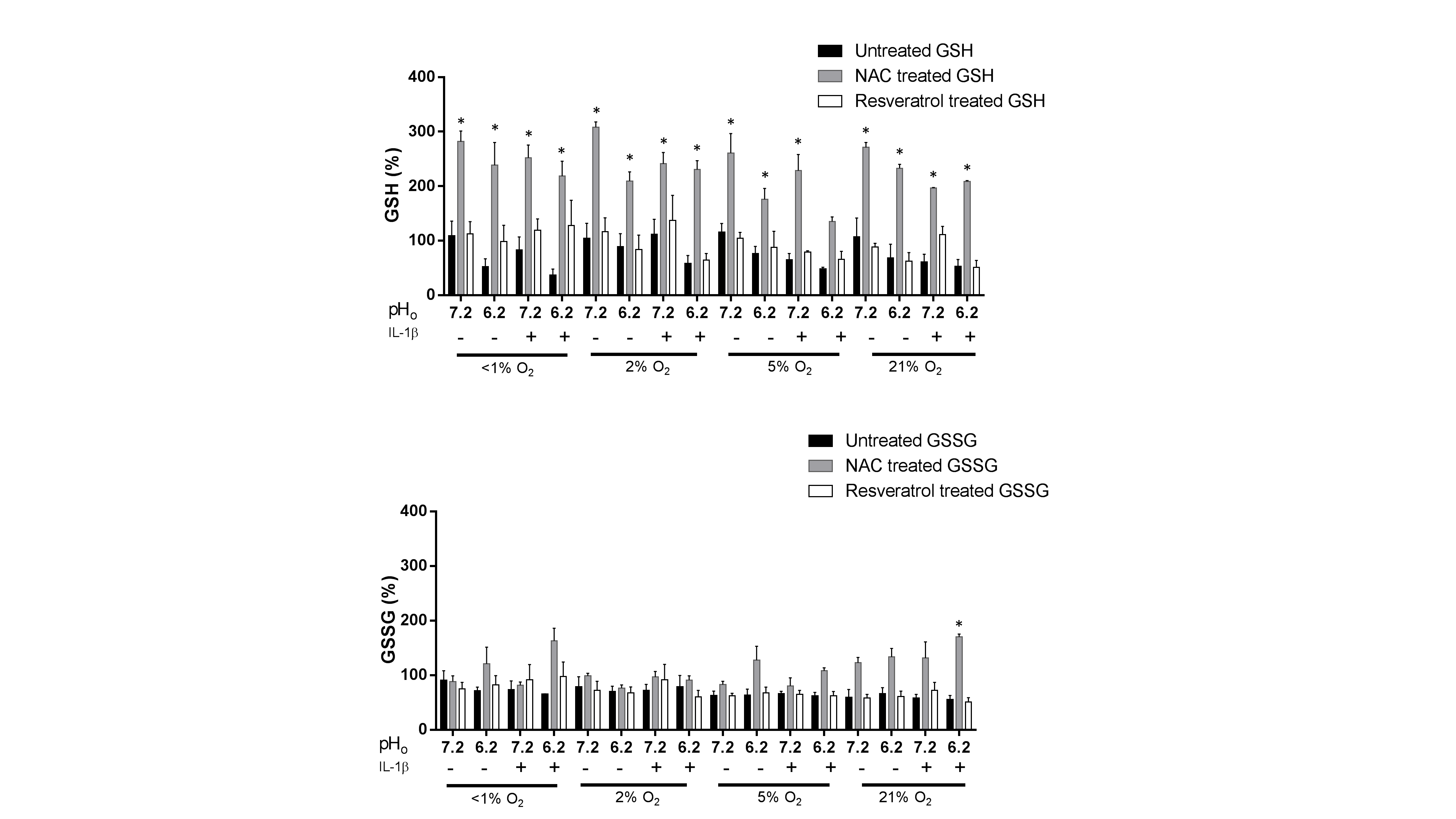

Supplement: Supplementary file 3 — Supplementary Material: Figure S3. (A) Reduced (GSH) and (B) oxidised glutathione (GSSG) levels in equine articular chondrocytes. Equine articular chondrocytes were cultured in 3D-alginate beads for 48 hours in <1%, 2%, 5% or 21% O2 at pH 7.2 or pH6.2 in the presence or absence of 10 ng/ml IL-1β and in the presence of resveratrol (10 µM) or N-acetylcysteine (2 mM). Data are presented as percentage luminescence of control (time = 0, 5% O2, pH 7.2). Bar charts represent mean ± SEM, n=3.*P<0.05 versus control. [file mmc3.zip › Figure S3 GSH GSSG.tif]

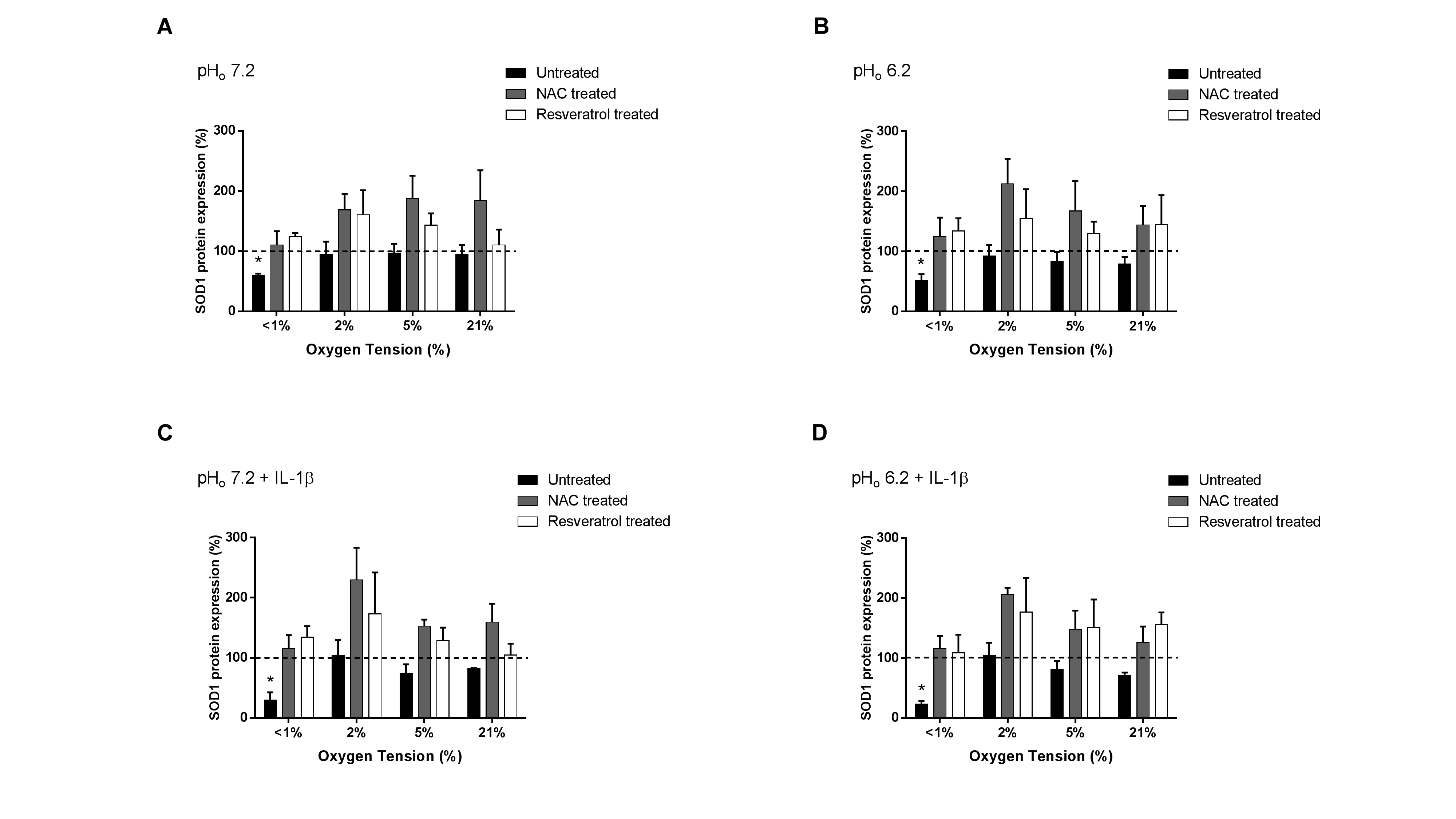

Supplement: Supplementary file 4 — Supplementary Material: Figure S4. Densitometric analysis of SOD1 protein expression from equine articular chondrocytes. Equine articular chondrocytes were cultured in 3D-alginate beads for 48 hours in <1%, 2%, 5% or 21% O2 at pH 7.2 (A) or pH6.2 (B) in the presence or absence of 10 ng/ml IL-1β (C, D) in the presence of resveratrol (10 µM) or N-acetylcysteine (2 mM). Data are normalised to α-tubulin and expressed as percentage of control (time = 0, 5% O2, pH 7.2). Bar charts represent mean ± SEM, n=3.*P<0.05 versus control. [file mmc4.zip › Figure S4 SOD1 densitometry.tif]

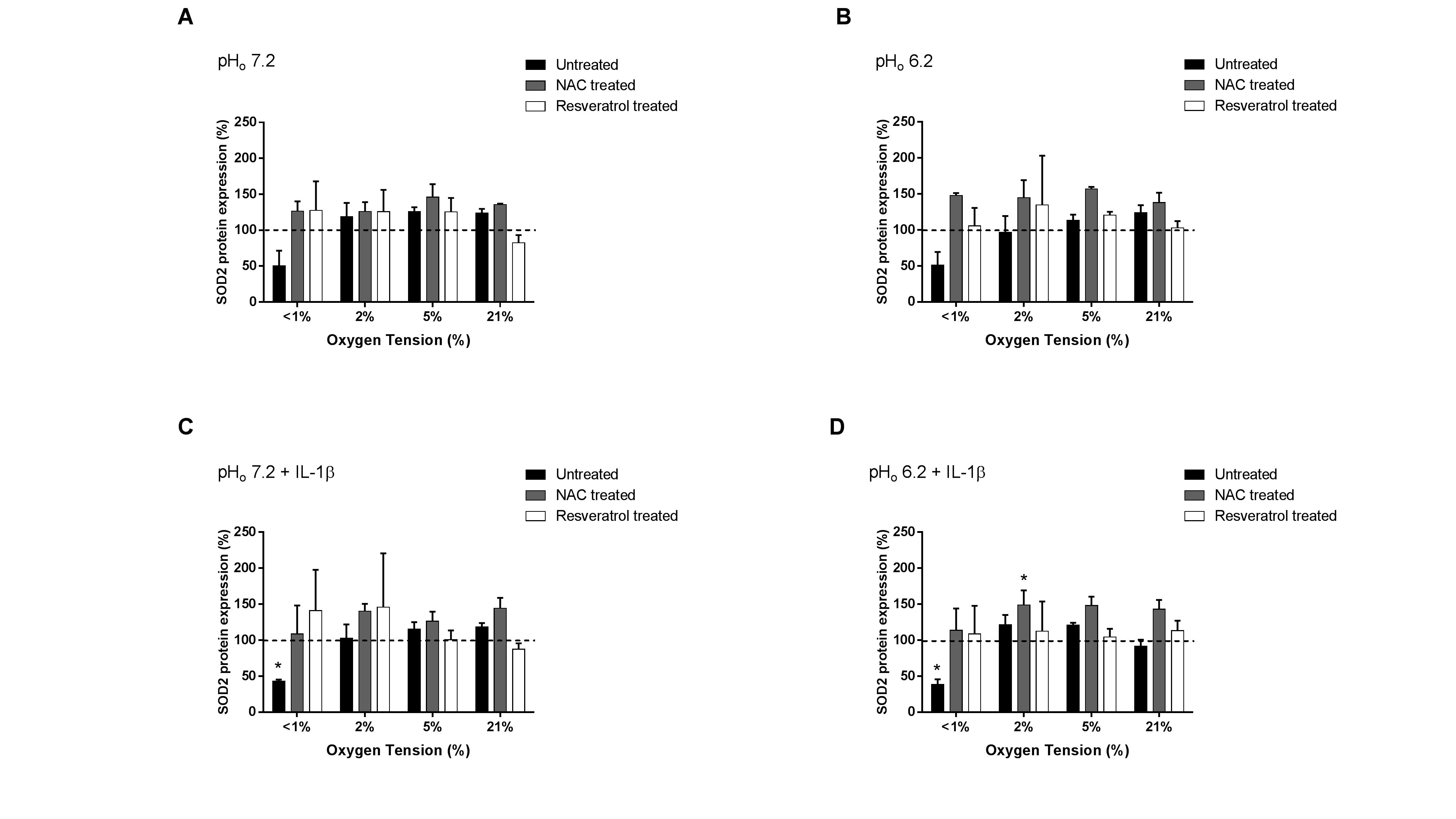

Supplement: Supplementary file 5 — Supplementary Material: Figure S5. Densitometric analysis of SOD2 protein expression from equine articular chondrocytes. Equine articular chondrocytes were cultured in 3D-alginate beads for 48 hours in <1%, 2%, 5% or 21% O2 at pH 7.2 (A) or pH6.2 (B) in the presence or absence of 10 ng/ml IL-1β (C, D) in the presence of resveratrol (10 µM) or N-acetylcysteine (2 mM). Data are normalised to α-tubulin and expressed as percentage of control (time = 0, 5% O2, pH 7.2). Bar charts represent mean ± SEM, n=3.*P<0.05 versus control. [file mmc5.zip › Figure S5 SOD2 densitometry.tif]
